# The Detrimental Effect of Thyroiditis on Pregnancy Outcome of Patients Affected by Autoimmune Diseases: An Open Question

**DOI:** 10.3389/fphar.2022.827735

**Published:** 2022-02-09

**Authors:** Angela Botta, Francesca Rizzo, Tatiana Antonielli, Alessandra Ciliberti, Ester Garufi, Antonio Lanzone, Cristina Garufi, Sara De Carolis

**Affiliations:** ^1^ Dipartimento Scienze della Salute della Donna, Del Bambino e di Sanità Pubblica, Fondazione Policlinico Universitario A. Gemelli IRCCS, Rome, Italy; ^2^ Dipartimento Scienze della Salute della Donna, Del Bambino e di Sanità Pubblica, Fondazione Policlinico Universitario A. Gemelli IRCCS, Università Cattolica Del Sacro Cuore, Rome, Italy; ^3^ Department of Anesthesiology and Intensive Care Medicine, Fondazione Policlinico Universitario A. Gemelli IRCCS, Roma, Italy; ^4^ Arthritis Center, Reumatologia, Dipartimento di Scienze Cliniche Internistiche, Anestesiologiche e Cardiovascolari, Sapienza Università di Roma, Roma, Italy

**Keywords:** thyroiditis, pregnancy outcome, autoimmune diseases, connective tissue disease (CTD), major rheumatic diseases (MRD), systemic lupus erythematosus (SLE), antiphospholipid syndrome (APS)

## Abstract

Few data are available evaluating obstetrical outcome when thyroiditis coexist with autoimmune diseases. Objectives of our study were: 1) To assess the prevalence of thyroiditis in pregnant women with autoimmune diseases; 2) To evaluate the effects on pregnancy outcome when different autoimmune diseases are associated with thyroiditis. Two groups of pregnant women were analysed: a study group of pregnant women with autoimmune diseases (*n* = 268) *versus* a control group of pregnant women (*n* = 1,150). In both groups the research for thyroid antibodies, anti-thyroid peroxidase antibodies and anti-thyroglobulin antibodies, was performed. The positivity had a prevalence of 17.54% in women with autoimmune diseases (*n* = 47) *versus* 5.57% in the control group (*n* = 64) (*p*-value < 0.00001). Only major rheumatic diseases (MRD) were analysed for pregnancy outcome (week of delivery, birth weight and birth weight percentile): systemic lupus erythematosus (SLE) *n* = 36, antiphospholipid syndrome (APS) *n* = 44 and connective tissue diseases (CTD) *n* = 23. MRD were divided according to positive or negative results for thyroid antibodies. Thyroiditis in CDT patients showed a detrimental effect on pregnancy outcome, in terms of earlier week of delivery: 37.86 ± 0.90 (mean ± SD) in CTD with thyroiditis *versus* 38.56 ± 0.73 (mean ± SD) in CTD without thyroiditis (*p*-value = 0.03) and lower birth weight: 2,790.71 g ± 257.17 SD in CTD with thyroiditis *versus* 3,019.33 g ± 305.48 g in CTD without thyroiditis (*p*-value < 0.05). In SLE and APS thyroiditis did not appear to influence pregnancy outcome. However, we suggest investigating anti-thyroid antibodies in all autoimmune diseases with special attention to pregnant women with thyroiditis and CTD.

## Introduction

During pregnancy, the maternal thyroid gland faces several metabolic, hemodynamic, and immunologic changes (Gaberšček and Zaletel, 2011). In particular, the presence of thyroid antibodies against thyroglobulin (anti-TG), thyroid peroxidase (anti-TPO), or thyrotropin receptor autoantigens (anti-TR) are common pregnancy-related diseases. The prevalence of thyroid peroxidase antibodies is increased almost 10-fold in women compared with men, it increases with age, and it has been reported in 2.7–10% of pregnant women (Pop et al., 2003; Casey et al., 2007; Negro et al., 2011; El Baba and Azar, 2012). Therefore, it is significant to track pregnant women with hypothyroidism, who consider pregnancy, furthermore treatment has demonstrated to improve implantation rate and live birth rate in sub fertile women (Myneni et al., 2021). A useful algorithm was recently proposed by expert opinions for the assessment and management of thyroid diseases in the pre-conception period or early pregnancy (Anandappa et al., 2020). The screening for thyroid diseases is highly recommended in case of women with autoimmune conditions (Anandappa et al., 2020).

In fact, autoimmune thyroid disorders are frequently associated with other organ and non-organ-specific autoimmune diseases, especially in major rheumatic diseases (MRD), such as systemic lupus erythematosus (SLE), antiphospholipid syndrome (APS) and connective tissue diseases (CTD) (Tagoe, 2015).

Many reports in the last decades documented the deleterious impact that thyroid disease has on pregnancy and postpartum period in terms of spontaneous abortion, prematurity, gestational diabetes, low birth weight (Saki et al., 2014) or large birth weight and placental weight (Tagoe, 2015), increased perinatal mortality (Männistö et al., 2009) preterm delivery (Abbassi-Ghanavati et al., 2010; Haddow et al., 2010; Stagnaro-Green et al., 2011; Korevaar et al., 2019) and postpartum thyroiditis. Particularly, thyroid antibody positivity in euthyroid women have been associated with miscarriage and preterm delivery (Stagnaro-Green, 2009).

Although either thyroid or rheumatic autoimmune disorders have been associated with pregnancy complications, there are scarce data in literature on the effect of the association between the two disorders on pregnancy outcome.

The pathogenic mechanisms of this coexistence are not completely defined, but genetics, epigenetics, immune defects, hormonal and environmental factors may play pivotal roles in poly-autoimmunity.

The objectives of our study were: 1) To assess the prevalence of autoimmune thyroiditis in pregnant women affected by autoimmune diseases in comparison to that of control pregnant women; 2) To evaluate the effects on pregnancy outcome when different autoimmune diseases are associated with thyroiditis in terms of gestational week at delivery, birth weight and birth weight percentile.

## Methods

A retrospective observational study was performed, analysing two groups of pregnant women, consisted by a study group of pregnant women with autoimmune diseases (*n* = 268) *versus* a control group of control pregnant women (*n* = 1,150).

**FIGURE 1:**
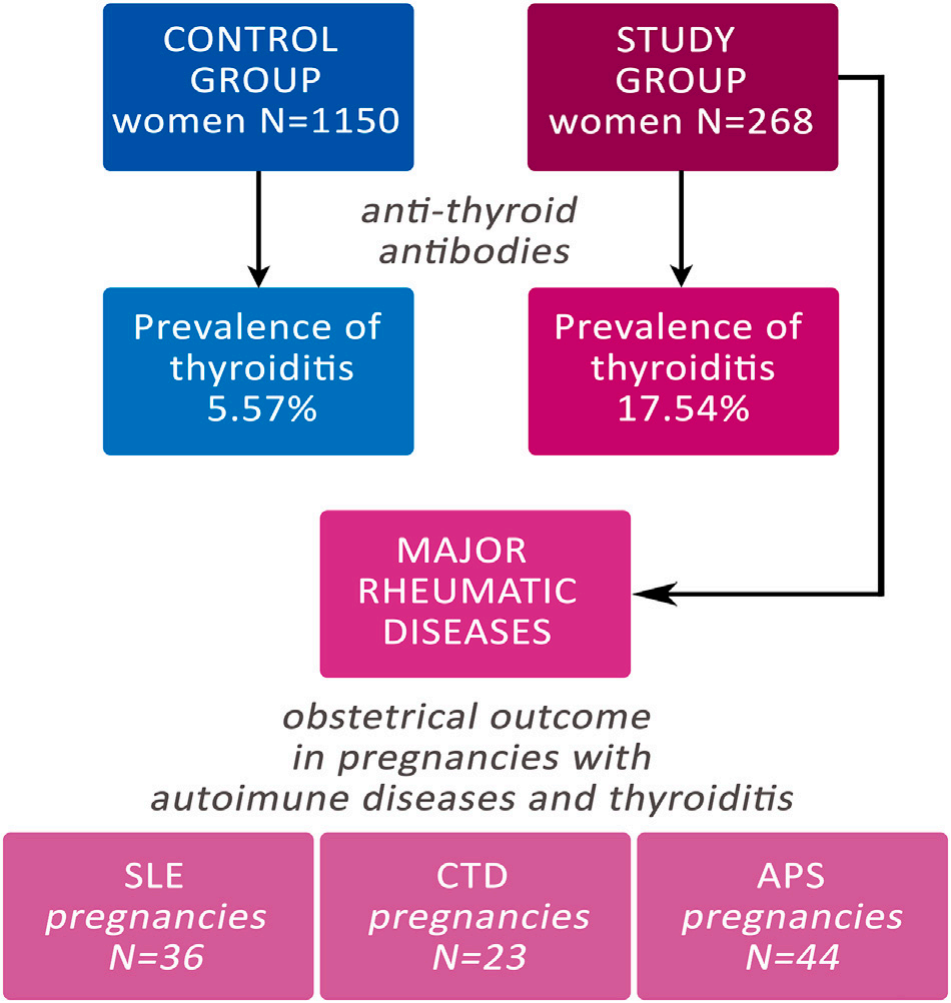
Flow-chart of the study. CTD: Connective Tissue Diseases; SLE: Systemic Erythematosus Lupus; APS: Anti-Phospholipid Syndrome.

The study group comprised 268 women with a confirmed diagnosis of autoimmune disease that were followed in our clinic throughout pregnancy and delivered in our centre from January 2010 to September 2020 (Fondazione Policlinico Universitario A. Gemelli—IRCCS). On the other hand, the control group comprised 1,150 pregnant women that consecutively delivered in our centre from September 2018 to December 2018 that included both physiological and complicated pregnancy (Figure 1).

Both in the study group and the control group, the research for thyroid antibodies, anti-TPO and anti-TG, was performed during the pregnancy, despite TSH value. In fact, the research involved either euthyroid pregnant women or women with altered thyroid function. In our series of cases, therapy of hypothyroidism was given/adjusted when necessary.

Patients with autoimmune diseases were 268 with an overall amount of 294 pregnancies, because some patients had more than one pregnancy in the described time interval. The study group was very heterogeneous, represented pregnancies with APS, SLE, CTD, arthritis, celiac disease, inflammatory bowel disease, Sjogren syndrome and other autoimmune diseases. Some patients had one or more autoimmune diseases.

The analysis was subsequently focused on pregnancies of patients suffering for major rheumatic diseases (MRD): SLE, APS and CTD. To compare the pregnancy outcome in the two groups (study group *versus* control group), multiple pregnancies and intrauterine fetal deaths were not included in the analysis. Therefore, pregnancies with MRD eligible for the analysis were: SLE *n* = 36, APS *n* = 44, and CTD *n* = 23. The three different disorders considered in MRD were divided according to the positive or negative results for thyroid antibodies.

Furthermore, the obstetric outcome was analysed in terms of week of delivery, neonatal birth weight, and neonatal birth weight percentile for understanding the eventual detrimental role of thyroid antibodies in pregnant women having associated other autoimmune diseases.

For the evaluation of neonatal birth weight percentile an Italian population-based study was employed (Ferrazzani et al., 2017).

## Results

In the study group of women with autoimmune diseases 47 out of 268 resulted positive for thyroiditis having positive the research for anti-TPO, anti-TG or both antibodies, showing a prevalence of 17.54%. Out of 1,150 women of control group, 64 resulted positive for thyroid antibodies, with a positivity rate of 5.57%. The prevalence of thyroiditis was statistically significant increased in the study group (*p* < 0.00001) (Table 1).

**TABLE 1:** Prevalence of thyroiditis in the Study group of pregnant women with autoimmune diseases and in the Control group of pregnant women.

	Pregnant women (*N*)	Pregnant women with thyroid antibodies positivity (*N*)	Prevalence of thyroiditis
Study group	268	47	17.54%
Control group	1,150	64	5.57%
*p* value	—	—	<0.00001

In the study group the most common autoimmune disease associated with thyroiditis was celiac disease with a rate of 53% of thyroiditis. APS showed a rate of 35%, CTD a rate of 25%, and SLE had a rate of 24%.

The analysis of pregnancy outcome in the MRD group, according to the presence of autoimmune antibodies, revealed the following findings. Autoimmune thyroiditis in patients having CTD showed a detrimental effect on pregnancy outcome, in terms of earlier week of delivery: 37.86 ± 0.90 (mean ± SD) in CTD with thyroiditis *versus* 38.56 ± 0.73 (mean ± SD) in CTD without thyroiditis (*p* value = 0.03) and lower birth weight: 2,790.71 g ± 257.17 SD in CTD with thyroiditis *versus* 3,019.33 g ± 305.48 g in CTD without thyroiditis (*p* value < 0.05), and lower birth weight percentile, although the decrease did not reach a statistically significant difference.

No statistically significant difference in pregnancy outcome was reported in the other two groups (SLE and APS) when thyroiditis was associated (Table 2).

**TABLE 2:** Obstetrical outcome in pregnancies with thyroiditis associated with MRD (Major Rheumatic Diseases). CTD: Connective Tissue Diseases; SLE: Systemic Erythematosus Lupus; APS: Anti-Phospholipid Syndrome.

	Week of delivery Mean ± SD	Birth weight (g) Mean ± SD	Birth weight percentile Mean ± SD
CTD with thyroiditis	37.86 ± 0.90	2,790.71 ± 257.17	34.14 ± 30.89
CTD without thyroiditis	38.56 ± 0.73	3,019.33 ± 305.48	37.93 ± 17.09
*p* value	**0.03**	**<0.05**	0.36
SLE with thyroiditis	38.30 ± 1.83	3,050.50 ± 485.77	51.63 ± 27.09
SLE without thyroiditis	37.69 ± 2.07	2,867.39 ± 554.21	44.26 ± 25.07
*p* value	0.21	0.26	0.37
APS with thyroiditis	37.75 ± 2.56	2,834.58 ± 741.34	39.50 ± 29.62
APS without thyroiditis	36.55 ± 3.33	2,788.94 ± 730.03	50.13 ± 28.03
*p* value	0.12	0.42	0.12

The bold values represent the statistically significant results.

## Discussion

Autoimmune diseases could be considered a family of disorders that often coexist in the same subject. The isolated thyroiditis *per se* could impair the pregnancy outcome and fecundity in childbearing women. Up to date, only few data are available evaluating the obstetrical outcome when thyroiditis coexists with autoimmune diseases.

Miscarriage and preterm delivery are the most common obstetric complications in pregnant women with isolated thyroiditis, also in presence of a normal thyroid hormone status (Thangaratinam et al., 2011). It is still unclear whether thyroid antibodies exert a direct pathogenetic effect at the fetal-maternal interface or they represent an epiphenomenon of other autoimmune disorders underlying.

Concerning the impact on pregnancy outcome when thyroiditis is associated with autoimmune disorders, the results of the various studies are conflicting and non-conclusive (De Carolis et al., 2004; Stagnaro-Green et al., 2011; Beneventi et al., 2016; Cellini et al., 2020).

Our study is one of the few reports evaluating the impact of thyroid antibody positivity on the pregnancy outcome in women affected by autoimmune diseases. Two major considerations can be done: the first one is that the prevalence of thyroiditis was significantly increased in the autoimmune disease pregnant women (17.54%) respect to that of the control group (5.57%); the second one is that the coexistence of thyroiditis with autoimmune diseases negatively impaired the pregnancy outcome in a specific group of rheumatic disorders such as CTD.

Regarding the prevalence of thyroid antibodies in MRD pregnancies, our findings confirm prior reports. In fact, the prevalence of thyroiditis in pregnant patients with SLE was of 24% in our study, very similar to the findings by Stagnaro et al. (23.8%). These authors described also a very high rate of preterm delivery (67%) in women who had thyroid disease, compared with the SLE women that were thyroid disease free (18%) (Stagnaro-Green et al., 2011).

In women with primary APS and history of recurrent spontaneous abortions, it was described a prevalence of 27% of thyroid antibody positivity (De Carolis et al., 2004). Further, anti-thyroid positivity was often associated with either reduced fecundity or with poor pregnancy outcome (De Carolis et al., 2004). In our study, we confirm similar findings (35%) in pregnant women with APS, while we were not able to demonstrate an impact on pregnancy outcome in case of anti-thyroid antibody positivity.

Moreover, we observed in CTD patients a prevalence of 24% of thyroiditis, although in another study the overall prevalence of either anti-TPO or anti-TG detection was up to 62.3% among CTD patients while it was 8% among controls (Beneventi et al., 2016). Furthermore, these authors suggested that thyroid antibody positivity could increase the risk of adverse pregnancy outcome, in terms of spontaneous abortion, fetal growth restriction, preeclampsia, and preterm delivery (Beneventi et al., 2016).

Surprisingly, in our study, the presence of anti-TPO and anti-TG antibodies was related with an increased risk of adverse pregnancy outcome, in terms of a lower week of delivery and a lower birth weight only in patients with CTD. Probably, in cases with SLE and APS the results were compromised by the small sample size. Other weaknesses of our analysis were: the retrospective design of the study; the lack of informations about replacement treatment; and the absence of cases with miscarriages, because we recruited women after the earlier weeks of pregnancy.

Future larger studies need to clarify the role of thyroid antibodies in the different autoimmune diseases, and the eventual role of thyroid hormone replacement on pregnancy outcome.

Finally, it is well known the EULAR recommendations (Andreoli et al., 2017) are to investigate the thyroid diseases at pre-conception counselling as well as in pregnancy in SLE and APS patients.

In our opinion, it is important to extend the prenatal screening for thyroid antibodies to all women affected by autoimmune diseases, paying special attention to the patients with CDT. On the other hand, it could be a field of interest to do the screening for autoimmune diseases in women in childbearing age with thyroiditis, particularly in presence of new specific symptoms or rheumatic clinical manifestations.

Defining the co-morbidities in high-risk pregnancies, as are women with autoimmune diseases, could better stratify the risk profile of the patient and could lead the clinicians to a tailored management of pregnancy.

## Data Availability

The datasets presented in this article are not readily available due to the patients privacy. Requests to access the datasets should be directed to frarizzo92@gmail.com.
